# Complete Genome Sequence of *Streptococcus iniae* YSFST01-82, Isolated from Olive Flounder in Jeju, South Korea

**DOI:** 10.1128/genomeA.00319-15

**Published:** 2015-04-23

**Authors:** Sasikumar Rajoo, Wooyoung Jeon, Kyungmoon Park, Sungsik Yoo, Injung Yoon, Hongweon Lee, Jungoh Ahn

**Affiliations:** a Biotechnology Process Engineering Center, KRIBB, Daejeon, Republic of Korea; b University of Science and Technology (UST), Daejeon, Republic of Korea; c Department of Biological and Chemical Engineering, Hongik University, Seoul, Republic of Korea; d Choong Ang Vaccine Laboratory, Daejeon, Republic of Korea

## Abstract

*Streptococcus iniae* is associated with morbidity in commercial fish species, especially in olive flounders (*Paralichthys olivaceus*), and was recently identified as an emerging human pathogen. Here, we report the complete 2.09-Mb genome sequence of *S. iniae* strain YSFST01-82, isolated from an olive flounder with streptococcosis disease in Jeju, South Korea.

## GENOME ANNOUNCEMENT

*Streptococcus iniae* is a hemolytic Gram-positive coccus that was initially isolated in 1976 from skin abrasions on Amazon freshwater dolphins (*Inia geoffrensis*) in aquariums at San Francisco, CA, and New York, NY (1). The strain has been recognized as the causative agent of a highly contagious and fatal disease characterized by meningitis and panophthalmitis in fish (2). *S. iniae*–mediated streptococcosis has led to significant economic losses for fish farmers, particularly in the olive flounder (*Paralichthys olivaceus*), which is a major mariculture species in South Korea (3). Moreover, this strain might cause opportunistic infections in weakened or immunocompromised humans handling contagioned fish; hence, it may be considered an emerging zoonotic agent (4).

Whole-genome sequencing of *S. iniae* strain YSFST01-82, isolated from diseased olive flounder in Jeju, South Korea, was performed with 454 GS-FLX Titanium (Roche Diagnostics, Basel, Switzerland), optical restriction mapping (OpGen, Inc., Madison, WI), and the 3730XL DNA Analyzer (Applied Biosystems, Foster City, CA, USA). Using an initial round of shotgun pyrosequencing, the contigs were assembled by using the Newbler gsAssembler software version 2.5.3 (454 Life Sciences, Branford, CT) and were further combined with 3-kb paired-end reads down to 19 scaffolds. By using the restriction enzyme NheI, an optical map was generated, and the contig orientation was validated using the MapSolver software (OpGen, Inc.). An additional round of Sanger sequencing was necessary for complete gap closure and finishing. The PCR products were subjected to cycle sequencing with ABI BigDye Terminator version 3.1 and were analyzed using the 3730XL DNA Analyzer (Applied Biosystems). The Phred-Phrap-Consed program (5, 6) was used for sequence assembly and editing of the assembled sequences. Gene prediction was carried out using Glimmer version 3.02 (7) and the Clusters of Orthologous Groups (COG) and SEED databases (8); rRNA and tRNA genes were identified by utilizing RNAmmer version 1.2 (9) and tRNAscan-SE version 1.23 (10), respectively.

The genome of *S. iniae* is composed of 2,086,959 bp (36.8% G+C content). A total of 1,897 coding DNA sequences, 58 tRNA genes, and 15 rRNA genes were predicted. The complete genome sequence of the *S. iniae* found in olive flounder skin lesions will aid in the research of this strain and establish a basis for molecular evolution studies of its systemic invasion, which will lead to a more efficient vaccine and diagnostic methods to establish proper disease control measures.

### Nucleotide sequence accession number. 

This complete genome sequence of strain YSFST01-82 has been deposited at GenBank under the accession no. CP010783.
